# Early tracking and immigrant optimism: a comparative study of educational aspirations among students in disadvantaged schools in Sweden and the Netherlands

**DOI:** 10.1186/s40878-017-0063-1

**Published:** 2017-12-14

**Authors:** Olav Nygård

**Affiliations:** ISV-REMESO, Linköping University, 601 74 Norrköping, Sweden

**Keywords:** Educational tracking, Doxic aspirations, Habituated aspirations, Social capital, Immigrant optimism

## Abstract

Educational tracking affects both the trajectories and the composition of peers that students meet in school. This study compares the effect of significant others on students’ educational aspirations within two transition regimes: the more comprehensive Swedish system and the more stratified Dutch. Separating between doxic and habituated aspirations, I hypothesize that (1) aspirations among students in disadvantaged schools will be lower in the Netherlands than in Sweden; (2) the higher educational aspirations of girls and children of immigrants will disappear when significant others are controlled for; and (3) the positive effect of significant others is more marked among Swedish students than among Dutch due to greater student heterogeneity. The data comes from 3202 students in schools with low average grades in Sweden and the Netherlands. Results were in line with the hypothesis with one important exception. There was a marked difference in habituated aspirations but no difference in doxic aspirations between the Dutch and Swedish students. In conclusion, the findings suggest a) that early tracking systems creates a disconnect between students’ hopes and what they perceive as likely outcomes, and b) that the phenomenon termed “immigrant optimism” and “ethnic capital” reflects unequal access to social capital.

It is well-known that early tracking, or the sorting of students on ability, leads to higher educational inequality and creates stronger associations between social background and students’ results (Breen & Jonsson, 2005; Burger, 2016; Horn, 2009; Le Donne, 2014; Van de Werfhorst & Mijs, 2010). Still, the effect of tracking on educational aspirations has not received the same attention. This is unfortunate, since aspirations can have a profound impact on educational trajectories. Students from advantaged background are more likely to pursue higher education than disadvantaged peers with identical grades. This is known as the “secondary effect” of class on educational inequality (Boudon, 1974). The secondary effect could be understood as reflecting the perceived “natural” choice given a student’s starting situation and her social, and institutional surroundings.

Across all EU countries, having well-educated parents and being female reduces risk of early school leaving while being foreign born increases it (Lavrijsen & Nicaise, 2015). However, the increased risk of early school leaving for children of immigrants is mainly due to lower grades and not due to lower aspirations.

The 2015 PISA results show that Immigrant students across OECD countries are more than twice as likely as non-immigrant peers to perform below baseline proficiency in science (OECD, 2016). Yet, children of immigrants tend to exhibit high levels of educational aspirations. When other factors are controlled for, children of immigrants are found to be more likely to make educational transitions (Jackson, 2012; Jackson, Jonsson, & Rudolphi, 2012), more likely to favor general over vocational educations (Kilpi-Jakonen, 2011), and more likely to enter tertiary education (Kristen, Reimer, & Kogan, 2008). This phenomenon has been attributed to “immigrant optimism” (Feliciano & Lanuza, 2016; Fernández-Reino, 2016; Kao & Tienda, 1995), even though it is not exclusive to immigrant minorities. Similarly high levels of educational aspirations have for example also been reported for Asian American youth (Goyette & Xie, 1999), and non-white ethnic minorities in the UK (Modood, 2004).

In this article, I will examine the effects of early tracking and social context on educational aspirations by contrasting Sweden to the Netherlands, two countries with different educational systems and transition regimes (Tikkanen, Bledowski, & Felczak, 2015). Whereas the Swedish system is comprehensive until the beginning of upper secondary education, Dutch students are assigned academic tracks at the beginning of lower secondary education. This makes the Dutch educational system both more stratified and more selective than the Swedish (Jackson & Jonsson, 2013). If tracking does indeed shape aspirations, this contrast should make the effect clearly visible.

## Aim & research questions

In a 12-country comparison of educational aspirations,^1^ Buchmann and Dalton noted that “the effects of interpersonal influences on educational aspirations depend, in part, on the institutional contexts in which they function” (2002, p. 100). This suggests that a key mechanism in the formation of aspirations is the social surrounding, and that institutional features – such as the presence or absence of early tracking – influence students’ aspirations in part by sorting people in that context on certain characteristics, attitudes and behaviors. Consequently, the aim of this study is to examine the effects of early tracking and social contexts on students’ aspirations. I study the mechanisms outlined above by comparing the educational aspirations of Swedish and Dutch students in disadvantaged schools. Following this logic, I will also examine to what extent the so-called “immigrant optimism” is affected by these contexts. The article is structured around the following three questions

1. Are there differences in educational aspirations between Dutch and Swedish students in disadvantaged schools?
2. Are there differences in educational aspirations between children of different non/immigrant backgrounds in these two settings?
3. What are the effects of significant others on students’ educational aspirations?

## Background

The educational system influence behaviors by structuring their consequences. But it can also shape different social foci, influencing whom a person meets and interacts with (Feld, 1981). This means that the time of educational tracking affect students in two ways: first, by impacting the likelihood of different educational trajectories; and second by influencing the composition of peers with regards to social background, attitudes and academic outlooks. And both these factors, in turn, shape aspirations.

European educational systems differ quite markedly from each other. The Dutch school system contains several levels to which a student can enroll for secondary education: a general/academic track with three hierarchical levels and a vocational track with two. Since the academic tracks are considered more prestigious than the vocational, the result is a multi-tiered secondary education (Kalmijn & Kraaykamp, 2003). Sweden, by contrast, has a mainly comprehensive educational system, where selection is delayed until upper secondary school (Heath, Rothon, & Kilpi, 2008). The earlier tracking and more frequent transitions in the Dutch educational system means that students’ educational pathways are determined at a young age, with the transition from primary to lower secondary education is a defining moment in a person’s educational career (Stevens et al., 2014). The result is a higher degree of vertical stratification among students in secondary education in the Netherlands than the OECD average, whereas Sweden has a lower (OECD, 2013b). However, when it comes to equity between children of immigrants and their non-immigrant peers, test score gaps are much larger in Sweden than in the Netherlands (OECD, 2013a, 2016). Thus, while children of immigrants across Europe do worse than their non-immigrant peers, this is even more so in Sweden.

Both Swedish and Dutch schools are segregated along axes of ethnicity and migrant status. Still, there are differences in the migration histories of the two countries (OECD & EU, 2015). During the 1960s and 70s, both countries drew migration from southern Europe and the Mediterranean area. Largely motivated by economic factors, these migrants generally shared a low-education background. In addition, metropolitan Holland attracted migration from its (former) colonies in South America and South-East Asia. This migration was more varied in nature, with both political and economic driving factors, and consequently more varied socio-economically. Sweden instead attracted many migrants from neighboring Finland. Unlike the diverse Dutch (post)colonial migration, migration from Finland to Sweden was largely labor migration. In the last two decades of the 20th century, migration in both countries gradually changed. Still, the reasons for migration vary greatly among contemporary migrants to the two countries.

Between 2005 and 2013, the Netherlands saw relatively larger shares of migrants coming to work or as part of EU free movement, whereas Sweden had relatively larger proportion of immigrants entering through asylum or family reunification (OECD & EU, 2015). The circumstances surrounding migration are important, since they may result in selection both in the country of origin and the country of arrival (Belot & Hatton, 2012). To better account for this heterogeneity in this study, I will distinguish between children of immigrants from the EU and from Third Country, and between those born in Sweden and those who were not.

## Theoretical framework

When researching aspirations, scholars have noted a contemporary political discourse on education and work-life highlighting personal responsibility, ambition and entrepreneurship (Davies & Bansel, 2007; European Commission, 2004, 2006). Within this discourse, aspirations are championed as a “simple antidote for complex problems” (Stahl, 2014, p. 664) since aspirations are seen as contributing to both greater economic growth and equality of opportunity (Spohrer, 2011). The problem with this idea of a aspirational (Raco, 2009) or entrepreneurial (Dahlstedt & Hertzberg, 2014) citizenship, is that it individualizes and de-contextualizes the processes of social stratification in general, and educational aspirations in particular. This reduces aspirations to a psychological trait, often associated to other qualities like inspiration, self-esteem and self-efficacy (see Spohrer, 2011). In this article, I will instead argue that aspirations, like other attitudes and outlooks, are shaped by social and institutional surroundings. By doing so, I build on previous research (Buchmann & Dalton, 2002) by paying particular attention to gender and ethnicity – dimensions along which aspirations are commonly found to differ; and by separating between habituated and doxic aspirations.

### Habituated and doxic aspirations

The Wisconsin model stipulates that a person’s class background influences how teachers’, parents’ and friends’ will assess her, and that these significant others in turn influence what level of education and occupational status she aspires to (Sewell, Haller, & Portes, 1969). This association between socio-economic background and aspirations, mediated by significant others, is well documented (Madarasova Geckova, Tavel, Van Dijk, Abel, & Reijneveld, 2010; Marjoribanks, 1997, 2002, 2010; Sewell & Shah, 1968).

Research on aspirations commonly distinguish between idealistic and realistic aspirations (Haller, 1968), or between aspirations or expectations. However, to the extent that aspirations are formed through interactions with others, both aspirations and expectations will reflect beliefs about the reality rather than reality itself. From this starting point, Zipin, Sellar, Brennan, and Gale (2013) argue that idealistic aspirations and realistic expectations are better understood as reflecting a *doxic* and a *habituated* logic, respectively. The doxic logic is based on populist-ideological mediations, while the latter is based on biographical-historical conditions.^2^ This reconceptualization highlights that aspiration is a cultural, rather than personal, capacity (Appadurai, 2004). Doxic aspirations could thus be described as idealistic aspirations: they represent the level of education or career position that a person would ideally like to reach. Habituated aspirations, in turn, could be called realistic aspirations in the sense that they refer to the levels a person thinks she *can* reach. However, habituated aspirations aren’t necessarily “realistic” in a strict sense. The key insight following Boudon’s (1974) separation of primary and secondary effects in inequality of educational opportunity is that middle class students can attain low grades and still be more likely to pursue an academic career than working class students receiving better grades. Recognizing that a person’s aspirations are shaped by her habitus is thus a way of conceptualizing that what seems realistic to a person depends on her background, previous experiences and social context.

### Significant others and social capital

Significant others influence a person’s aspirations, their courses of actions and their beliefs about the world (Woelfel & Haller, 1971). However, aspirations are also shaped by material conditions. Taken together, the resources and aid that accrue to a person through her significant others can be understood as expressions of social capital. Following Bourdieu, Lin and others, social capital can be defined as resources embedded in networks and thus only available to a person through her interactions with others (Behtoui, 2006; Bourdieu, 1986; Flap & Völker, 2004; Lin, 2001). Using a social capital framework broadens the scope to not only include information, but also financial or emotional support, help with homework or using one’s name to vouch for someone. This emphasizes that norms, values and information can be complemented by more tangible resources, also drawn from the social network surrounding a person.

Among the most important sources or social capital for students are parents, peers, and teachers. Parents, influence educational outcomes and aspirations by engaging in different upbringing strategies, thereby reproducing privilege through cultural capital (Lareau, 2002). Peers’ attitudes and behaviors play important roles in shaping adolescents’ own plans and decisions (Flores-Gonzalez, 2005; Gabay-Egozi, Shavit, & Yaish, 2015; Willis, 1993 [1977]). British research has shown that students from low-income families with friends from high-income families were more likely to remain in education (Burgess & Umaña-Aponte, 2011). Teachers, finally, exert control over both own and institutional resources to aid – or hinder – students. Because of this, they both work within institutional constraints and have some possibility of working these constraints from within (Stanton-Salazar, 2010).

### Immigrant optimism?

Studies on educational outcomes of children of immigrants have found a recurrent asymmetry between grades, and educational choices (Heath & Brinbaum, 2014; Jackson, 2012; Jackson et al., 2012; Jonsson & Rudolphi, 2011; Urban, 2012). While children of immigrants receive lower average grades, they are more likely than their non-immigrant peers to pursue higher education and aspire for skilled professions. This is for example seen in PISA results, where immigrant students in both Sweden and the Netherlands are more likely to hold habituated aspirations – expectations – for careers in science than non-immigrant students, despite scoring lower test results in scientific ability. Of the 34 countries listed, the discrepancy between test scores and aspirations is largest and second-largest in Sweden and the Netherlands (OECD, 2016).

This recurrent finding has led researchers to speak of an “immigrant optimism” (Feliciano & Lanuza, 2016; Fernández-Reino, 2016; Kao & Tienda, 1995). What produces this optimism has however been debated. Recent research has found educational selectivity to be a key factor (Engzell, 2016; Ichou, 2014). Immigrants are not drawn randomly from the population of the country of origin. Rather, they are often positively selected on education, meaning that those who migrate are - on average - more well-educated than those who remain (Feliciano, 2005). Since educational systems differ, this distinction is lost when translated into years of education for comparison with persons in the destination country. Concretely, an immigrant with low educational qualifications relative to the average person in the country of destination may be highly qualified relative to those in the source country. This creates a mismatch between skills, reflected in absolute level of education, and cultural resources, values and outlooks based in the relative level of education. A lower absolute level of education among immigrants would thus result in a grade disadvantage for their children. Meanwhile, their high relative level of education means that they pass on higher levels aspirations and expectations. Educational selection thus impacts intergenerational mobility mainly through aspirations, not transfer of skills (Engzell, 2016).

Accounting for the relative level of education for parents only partly explains immigrant optimism. Rival explanations suggest that stronger academic preferences are part of a strategy to compensate for discrimination (Heath & Brinbaum, 2007; Urban, 2012), or stronger social networks in ethnic communities (Zhou & Bankston, 1994). In a classic study, Bankston and colleagues (Bankston, Caldas, & Zhou, 1997) found that the strongest predictor of Vietnamese students’ school achievements were the ethnic identities of their friends. Since then, a multitude of studies have found stronger educational norms and higher levels of aspirations among children of immigrants and other minorities (Archer & Francis, 2006; Brinbaum & Cebolla-Boado, 2007; D’hondt, Van Praag, Van Houtte, & Stevens, 2016; Goyette & Xie, 1999; Marjoribanks, 2002; Salikutluk, 2016; Teney, Devleeshouwer, & Hanquinet, 2013; Van de Werfhorst & Van Tubergen, 2007). Taken together, these results suggest the existence of an ethnic capital (Modood, 2004; Shah, Dwyer, & Modood, 2010), or rather ethnicized social capital.

Inequality in social capital arise from the fact that a person is more likely to interact with others in a similar status and class positions - for example colleagues and friends from school. This means that a working-class person can be expected to have more working-class acquaintances than a person from the middle class, allowing the latter better access to resources and information through her network. However, due to selection, the occupational position of an immigrant may not correspond well to her social status of class position in the country of origin. If this is true, the social networks of immigrants will be different than what could be expected from their occupational position. In other words, a person coming from a high-status background might retain her connections and thus the norms, values, help and information they provide, even when the material resources suffer from de-classing due to migration.

Furthermore, immigrants and their children are more likely to suffer from discrimination and exclusion than their non-immigrant peers (F. Ahmadi, Palm, I. & N. Ahmadi, 2016; Bursell, 2014; Verkuyten & Thijs, 2010). This makes perceived ethnic boundaries likely to shape interactions and thus network (re)formation. Since network connections facilitate interactions, information flow and norm diffusion, a perceived ethnic boundary can have the opposite effect. Consequently, different access to social capital between immigrants and non-immigrants likely depend on two factors. First, that social networks are not declassed through migration in the same way as occupational positions often are. And second, that ethnicized social exclusion makes it more difficult to enter new social relations with members of the majority. Because of this, the social capital of immigrants can be understood as *ethnicized*, or structured by a process of difference-making through perceived ethnicity. This means that minority youth might be drawing from a broader range of significant others, including a wider range of different class and occupational positions (Crozier & Davies, 2006).

Institutional arrangements have been found to affect the educational gaps between migrant and non-migrant students (Crul, 2013; Crul & Vermeulen, 2003). Research points both towards students with migrant background and low-educated parents faring worse (Griga & Hadjar, 2013) and better (Bauer & Riphahn, 2013) than non-migrant peers in systems with earlier tracking. This disparity between results point to the twin processes involved in shaping aspirations. Advantageous socio-economic background and the support from significant others are both commonly associated with higher levels of aspirations, and whereas migrant families may have less of the former, they may have more of the latter (Feliciano & Lanuza, 2016; Thapar-Bjorkert & Sanghera, 2010).

### Hypotheses

Drawing on this theoretical understanding, three hypotheses can be formulated.

1. That aspirations among students in disadvantaged schools will be lower in the Netherlands than in Sweden due to earlier tracking.
2. That the higher educational aspirations of girls and children of immigrants will disappear when significant others are controlled for.
3. That the positive effect of significant others is more marked among Swedish students than among Dutch.

## Data and variables

Data was drawn from a 2014 project on preventing early school leaving in Sweden and the Netherlands (Behtoui, 2017). Consequently, all students included attended schools with low average grades and a higher-than-average risk of students not finishing upper secondary education. Since educational aspirations are generally high (Goyette, 2008), this is a fruitful sampling strategy as it can be expected to produce greater variation in aspirations than probability sampling would. From a total of 4160 respondents aged 15–20, 958 were excluded due to incomplete data. This left 3202 cases, as outlined below.

T-test comparisons between excluded and included observations revealed that more male than female respondents had been excluded. Furthermore, children of immigrants from third country were also disproportionately excluded while a higher proportion of non-immigrant students were kept. As boys and students from third country tend to be disadvantaged in school, there may exist a correlation between marginalization and exclusion from the data set.

Variables were coded as follows:

*Doxic aspirations* was coded 1 if the student answered university or higher education on the question “what level of education would you like to reach before you start working”, while *Habituated aspirations* was coded 1 if the student answered that it was very likely that she would attain this level. The two measures displayed a correlation of 0.39.

*Grades* reflect last year’s average grades, ranging from 1 to 5. *High Educated Parents* was coded 1 for students with at least one parent having completed higher academic education, (ISCED 5A or 6) and 0 for all others, including cases where parents’ education is unknown.^3^

Five categories of children of immigrants were created based on the background of student’s families. Both Sweden and the Netherlands are part of the EU, giving citizens of one member state the right to travel, work and study in other member states. This freedom of movement potentially impacts the selectivity of migrants. A distinction was consequently made between children of immigrants from Europe and those from third country.^4^

Children of immigrants were in turn separated on whether the student was born outside of the country of survey or not. Students born in the country of survey were categorized as having immigrant background if both their parents were foreign born. If both parents were born outside of Europe, they were classified as having *TC-parents* (foreign background, parents from third country), otherwise as *EU-parents* (foreign background, parents from Europe). Finally, children of immigrants born outside of the country of survey were classified as *TC-born* (foreign born, from third country) or *EU-born* (foreign born, from Europe) depending on their regions of birth.

*Parents’ expectations* was coded 1 if the student reported that her parents expected her to go to university, while *teachers’ expectations* was coded similarly based on perceived expectations from teachers.^5^

*Peer study-attitudes* and *Peer adult-life attitudes* indicate the study oriented and adult-life oriented attitudes, respectively, among peers. The variables were created as factor composites (principal component, varimax) of seven Likert items (alpha 0.87) of perceived attitudes and aspirations among peers. The former loaded primarily on items such as “My friends think it is important to study to get a good future” whereas the latter loaded on items indicating that friends found it important to earn their own money or start a family.

*Female friends* is the respondent’s perception of the proportion of same-gendered friends, reversed for male students to form a unified measure of (perceived) proportion of girls in peer group.^6^ Finally, *NL* indicates whether a student was surveyed in the Netherlands, and thus had experienced early tracking, or not.

The descriptive data (Table 1) show a slight overweight of Dutch respondents. Students surveyed in disadvantaged schools in the Netherlands held lower average aspirations, both habituated and doxic. Their parents in turn were to a lower degree university educated. These differences are in line with the expected results of tracking, with the stronger selection of Dutch students in the sample resulting in a more homogenously marginalized group.

**Table 1:** Descriptive statistics, respondents from the Netherlands and Sweden

Variable	The Netherlands	Sweden	Min	Max
	Mean (std. d.)	Mean (std. d.)		
Doxic aspirations	.4591652 (.4984805)	.6229826 (.4847958)	0	1
Habituated aspirations	.0931639 (.29075)	.2143318 (.4104905	0	1
Grades	3.692075 (.9768092)	2.488702 (1.071432)	1	5
HE parents	.2020569 (.4016559)	.29051 (.4541444)	0	1
Female	.5595886 (.4965867)	.4887024 (.5000338)	0	1
Non-migrants	.4010889 (.4902673)	.5216269 (.4996934)	0	1
EU-parents	.0205687 (.1419781)	.1000646 (.300183)	0	1
EU-born	.015729 (.1244626)	.0548741 (.2278079)	0	1
TC-parents	.4500907 (.4976534	.1969012 (.3977855)	0	1
TC-born	.1125227 (.3161041)	.1265332 (.3325567)	0	1
Age	16.96007 (1.235415)	16.58425 (.7634478)	15	20
Parents’ expectations	.1506352 (.3578012)	.5745642 (.4945686)	0	1
Teachers’ expectations	.2728373 (.4455527)	.3660426 (.4818769)	0	1
Peer study-attitudes	−.1909591 (.9689289)	.3268179 (.8611838)	−4.088771	1.834592
Peer adult-life attitudes	.1555228 (.8510057)	−.1955836 (1.113068)	−3.291466	2.352939
Female friends	3.113128 (1.220746)	2.92705 (1.318904)	1	5
N	1653	1549		

There are also marked differences in the composition of students surveyed in the two countries. The colonial past is seen in the Dutch cohort having a larger proportion of children of immigrants from third country, whereas the Swedish has relatively more children of immigrants from Europe. Due to these differences, some categories became very small with first and second-generation European migrants comprising approximately 3, 5% of the surveyed students in the Netherlands.

## Method

Since students naturally attend different schools in different areas, random-intercept multi-level regressions were fitted. This models a situation where individuals vary around a group mean whereas groups vary around a general mean, factoring in that selection and other unobserved factors makes students from a single school more similar than students selected at random. However, as no group level variables are included in the data, no second level predictors are estimated. Instead, the multi-level models are only used to account for these naturally occurring clusters without losing power. To investigate robustness, single-level logit models and single level OLS regressions using clustered standard errors were also fitted. Unless otherwise noted, the complementary models produced similar estimates while likelihood ratio tests indicated superior model fit for the multilevel-models. Results from the complementary models are available upon request.

## Results

To study the effects of early tracking on students’ aspiration, regressions were fitted both on pooled data and separated by country. The results are presented below.

Table 2 shows that the main co-variants of university aspirations in both countries were the expectations of parents and teachers. Both estimates returned highly significant and with large coefficients. Peer study-attitudes and the proportion of female friends were associated with higher levels of university aspirations among Swedish students but not among the Dutch. This is consistent with hypothesis 3, predicting that significant others would be more important for shaping aspirations in the less-homogenous Swedish schools (Buchmann & Dalton, 2002).

**Table 2:** Multi-level logit regressions of doxic university aspirations

Variable	Total sample	The Netherlands	Sweden
Grades	0.273***	0.110	0.501***
HE parents	0.339**	0.558***	−0.041
Female	0.215	0.243	0.146
Non-immigrants (reference)			
EU-parents	−0.148	−0.421	−0.123
EU-born	0.160	0.890	0.047
TC-parents	0.315**	0.346*	0.385
TC-born	0.199	−0.072	0.804**
Age	−0.153**	−0.133*	−0.309**
Parents’ expectations	1.699***	1.170***	2.070***
Teachers’ expectations	1.423***	1.613***	1.127***
Peer study-attitudes	0.168***	0.094	0.299***
Peer adult-life attitudes	−0.057	−0.023	−0.074
Female friends	0.057	−0.016	0.184*
NL	−0.361		
Constant	0.588	0.650	2.219
Constant (Level 2)	0.526***	1.110*	0.075
N	3202	1653	1549

Legend: *p < .05; **p < .01; ***p < .001

To better assess the effects of significant others, stepwise regressions were performed separately for each country (see Table 3). In the first model for each country, only student characteristics were included. In the second model, peer groups were included, in the third teachers’ and parents’ expectations. The first model indicated a strong positive association between being female and having university aspirations for Dutch students. However, once peers were included this association disappeared. Similar results were also found in Sweden. Model 1 also indicated higher educational aspirations among EU-born, TC-born and TC-parents students in the Dutch cohort. These associations weakened with the inclusion of significant others into the regression; for all but TC-parents to the point of disappearing, even though the coefficients remained large for EU-born.^7^

**Table 3:** Multi-level logit regressions. The effects of significant others on Dutch & Swedish students’ doxic university aspirations

Variable	The Netherlands			Sweden		
	Model 1	Model 2	Model 3 (full)	Model 1	Model 2	Model 3 (full)
Grades	0.209***	0.201**	0.110	0.807***	0.783***	0.501***
HE parents	0.679***	0.686***	0.558***	0.252	0.279	−0.041
Female	0.293*	0.200	0.243	0.553***	0.309	0.146
Non-immigrants (reference)				–	–	–
EU-parents	−0.102	−0.110	−0.421	0.704**	0.656**	−0.123
EU-born	1.153*	1.125*	0.890	0.769**	0.702*	0.047
TC-parents	0.566***	0.538***	0.346*	1.057***	1.067***	0.385
TC-born	0.391*	0.367	−0.072	1.633***	1.590***	0.804**
Age	−0.129*	−0.137*	−0.133*	−0.392***	−0.378***	−0.309**
Parents’ expectations			1.170***			2.070***
Teachers’ expectations			1.613***			1.127***
Peer study-attitudes		0.174**	0.094		0.414***	0.299***
Peer adult-life attitudes		0.011	−0.023		−0.042	−0.074
Female friends		0.021	−0.016		0.087	0.184*
Constant	0.611	0.799	0.650	4.175*	3.759*	2.219
Constant (Level 2)	1.036**	1.036**	1.110*	0.295**	0.247*	0.075
N	1653	1653	1653	1549	1549	1549

Legend: *p < .05; **p < .01; ***p < .001

For the Swedish cohort, all categories of children of immigrants exhibited higher levels of aspirations in the first two models. Like in the Dutch cohort, the associations weakened or even disappeared with the inclusion of peers, teachers’ and parental expectations. However, a large positive effect for TC-born even in the final model. Robustness tests returned comparable results. Taken together, the findings from both Sweden and the Netherlands are largely consistent with the second hypothesis, indicating that some – or even most – of both the “immigrant optimism” and the stronger aspirations among girls is due to their significant others.

Aspirations among Swedish students were more dependent on their previous grades, whereas parents’ level of education was more important for the Dutch students. Yet, as Tables 2 and 3 show, there was no difference in overall university aspirations between the Swedish and the Dutch students. This contradicts the first hypothesis, which predicted that earlier tracking would have a negative impact on aspirations.

Turning instead to habituated aspirations (Table 4), the regression showed higher grades to be positively correlated with higher habituated aspirations. Again, however, expectations from parents and teachers were a major factor in both countries. In addition, the regressions also returned significant and positive coefficients for *peer study-attitudes*. Finally, there was also a negative association between age and habituated aspirations.

**Table 4:** Multi-level logit regressions. Habituated university aspirations

Variable	Total sample	The Netherlands	Sweden
Grades	0.637***	0.278**	0.838***
HE parents	0.350**	0.321	0.310
Female	−0.384*	−0.294	−0.451*
Non-immigrants (reference)			
EU-parents	−0.138	−0.259	−0.031
EU-born	−0.566	0.148	−0.619
TC-parents	−0.164	−0.263	0.005
TC-born	−0.000	−0.326	0.323
Age	−0.249**	−0.207*	−0.281*
Parents’ expectations	0.917***	0.933***	0.878***
Teachers’ expectations	1.043***	1.398***	0.794***
Peer study-attitudes	0.370***	0.283**	0.477***
Peer adult-life attitudes	0.007	−0.106	0.065
Female friends	0.156*	0.024	0.229**
NL	−0.908***		
Constant	−0.568	−0.497	−0.767
Constant (Level 2)	0.107*	0.157	0.016
N	3202	1653	1549

Legend: *p < .05; **p < .01; ***p < .001

Unlike the doxic aspirations – discussed above – there was also a negative and significant coefficient for NL. This means that students in the Dutch cohort were less confident that they would complete higher education than their Swedish counterparts, given the same grades and social background. This is consistent with the first hypothesis, and suggests different mechanisms for habituated and doxic aspirations.

Separate stepwise models were run for the Dutch and Swedish cohort to investigate any mediating effects that significant others might have (Table 5). As above, the first models included only student and family characteristics. The second models included peer groups, whereas the third included teacher and parent expectations. For the Dutch cohort, no significant difference existed between models 1 and 2. However, between models 2 and 3 the association between *Higher Education parents* and *Habituated aspirations* disappeared. This indicates that the expectations of significant others have a mediating effect for habituated aspirations as well, and that it mediates the effects of parents’ educational background or their cultural capital (Bourdieu, 1986).

**Table 5:** Multi-level logit regressions. The effects of significant others on habituated university aspirations for Dutch & Swedish students

Variable	The Netherlands			Sweden		
	Model 1	Model 2	Model 3	Model 1	Model 2	Model 3
Grades	0.393***	0.382***	0.278**	0.979***	0.985***	0.838***
HE parents	0.500*	0.518*	0.321	0.403**	0.421**	0.310
Female	−0.049	−0.298	−0.294	0.128	−0.329	−0.451*
Non-immigrants (reference)						
EU-parents	0.077	0.083	−0.259	0.478*	0.371	−0.031
EU-born	0.707	0.636	0.148	−0.147	−0.239	−0.619
TC-parents	0.060	−0.005	−0.263	0.499**	0.388*	0.005
TC-born	0.234	0.170	−0.326	0.780***	0.721**	0.323
Age	−0.229*	−0.232*	−0.207*	−0.319**	−0.296**	−0.281*
Parents’ expectations			0.933***			0.878***
Teachers’ expectations			1.398***			0.794***
Peer study-attitudes		0.375***	0.283**		0.583***	0.477***
Peer adult-life attitudes		−0.066	−0.106		0.057	0.065
Female friends		0.068	0.024		0.188*	0.229**
Constant	−0.094	−0.028	−0.497	0.795	−0.153	−0.767
Constant (Level 2)	0.239	0.245	0.157	0.020	0.018	0.016
N	1653	1653	1653	1549	1549	1549

Legend: *p < .05; **p < .01; ***p < .001

For students in the Swedish cohort (Table 5), the first model showed a significant positive association between TC-parents and Habituated aspirations. However, much like in the models on doxic aspirations (Table 3), this association disappeared with the inclusion of peers.^8^

Across the first three models, there was a positive association between having high-educated parents and habituated aspirations. However, this association disappeared in the full model, which again suggests a class aspect of educational expectations. Surprisingly, Swedish female students had lower habituated aspirations than their male counterparts. Students with more female friends on the other hand had higher habituated aspirations. Since girls are likely to have more female friends – and on average get better grades – these effects are likely to offset each other. And indeed, the estimates for female start out indicating no gender difference in models 1 and 2. This general trend is consistent with hypothesis 2.

## Discussion

This article has focused on the differences in educational aspirations produced by the Swedish and the Dutch educational system. Due to early educational tracking, students in the Netherlands face very different chances of attaining university credentials based on their performance at the end of primary school. In this sense, their futures are largely given by their past since social inheritance is often stronger in systems with earlier, and more consequential, choice points (Breen & Jonsson, 2005; Burger, 2016). Compared to students in the Swedish comprehensive educational system, Dutch students were significantly less likely to think they would succeed in attaining university education. As educational levels strongly influence the future labor market position of a person, lower habituated aspirations for education also means that students are more pessimistic about their future careers.

Yet, this pessimism is absent when students were asked what level of education they would like to achieve. This could be an indicator that high educational aspirations are increasingly becoming, or being promoted as, the norm (see Goyette, 2008; Spruyt, De Keere, Keppens, Roggemans, & Van Droogenbroeck, 2014). Even though the Dutch students in this study found it less likely that they would reach the desired educational outcome, they still exhibited as strong preference for university education as the Swedish students. Consequently, it appears that a key product of the Dutch early tracking on aspirations is a disconnect between doxic and habituated aspirations, or what students hope to achieve and what they think is within their capabilities.

The results also showed that parents and teachers had a large part in shaping students’ aspirations in both countries. Peer groups, on the other hand, had a considerable influence on the Swedish students’ aspirations, but only on the habituated aspirations of Dutch students. Swedish students, were also more influenced by the peer-group’s gender composition – a factor that largely correlates with high educational aspirations and good grades.

It has been argued that children of immigrants experience a disconnect between higher abstract and lower concrete educational aspirations in countries with stratified educational systems (D’hondt et al., 2016). This study, however, found no indication that this would be the case. Instead, migrant students and students with immigrant background reported higher average expectations from parents, and these expectations served to boost their own aspirations. This supports the hypothesis that stronger educational norms and cultures of aspirations can flourish in groups experiencing ethnicized exclusion or social closure, but suggests a causal mechanism in the effect of significant others.

Reflecting the separate migration histories of the two countries, the association between the various categories of children of immigrants and aspirations vary between Sweden and the Netherlands. Among Dutch students, only those with foreign born parents exhibited higher doxic aspirations. By contrast in Sweden, both foreign born students and students with foreign born parents exhibited higher levels of university aspirations than students with Swedish background when significant others were not accounted for. In both settings, these associations were reduced in strength – sometimes to the point of disappearing – once significant others were controlled for.

The higher levels of aspirations among students with migrant background have been described as “immigrant optimism” (Fernández-Reino, 2016), or conceptualized as “ethnic capital” (Modood, 2004; Shah et al., 2010). The results presented above indicate that this immigrant optimism might be better understood as reflecting unequal access to social capital. Based on earlier research I have argued that different access to social capital between immigrants and non-immigrants likely depend on two factors: that social networks are not declassed in migration in the same way as occupational positions are; and that ethnicized social exclusion makes it more difficult to enter new social relations with members of the majority. Because of this, the access to social capital of immigrants might be higher than suggested by their occupational position alone.

### Concluding remarks

This study compared educational aspirations between Dutch and Swedish students in disadvantaged areas. Three hypotheses were tested

1. That aspirations among students in disadvantaged schools will be lower in the Netherlands than in Sweden due to earlier tracking.
2. That the higher educational aspirations of girls and children of immigrants will disappear when significant others are controlled for.
3. That the positive effect of significant others is more marked among Swedish students than among Dutch.

Findings largely supported these hypotheses, with the exception that the earlier tracking seemed only to affect habituated – or “realistic” – aspirations of Dutch students. Further studies are needed to determine if similar processes apply to students in non-disadvantaged schools, but also to assess the psychological and social consequences of students’ attempts to reconcile their high educational ideals with the perceived hopelessness of making them come true.

## Footnotes

1 The countries included were Austria, France, Germany, Greece, Hong Kong, Hungary, Korea, Norway, Spain, Switzerland, Thailand, and the US.

2 Zipin, Sellar, Brennan, and Gale also conceptualize a third aspirational logic. This logic, they argue, should be understood as emergent and is based in lived socio-cultural resources or “funds of aspirations” (Zipin et al., 2013). Such aspirations are seldom verbalized as they follow no previously established blueprint. Rather, they are the intimation of unrealized futures. For this reason, they are difficult to research and must therefore be excluded from this study.

3 Models were also fitted including parents’ occupation. This further excluded 520 cases but otherwise produced similar results. For this reason, and because parents’ occupation can be assumed to correlate with parents’ education and grades which are both included in the models, the final models are here presented without parental occupation.

4 Due to data limitations, Europe is here used as a proxy for the EU free movement zone.

5 As both expectations, attitudes and own aspirations are reported by the student, it could be argued that they might all be colored by the student’s self-assessment. In other words, a student with high aspirations might think that her parents and teacher hold higher expectations than they do. While this is a relevant critique, the focus of this article is not the exact effect sizes but the comparison between Dutch and Swedish educational systems. Since data was collected in similar fashion in both countries, there is no reason to believe that any systematic error biased this comparison.

6 Models were also fitted with the original variable, both for all students and separately by gender. Results showed a positive effect (*p* < 0.1) of having same-sex friends for women and a neutral or negative effect (*p* < 0.001) for men.

7 In robustness tests with OLS regressions, the association between TC-parents and doxic university aspirations also disappeared fully with the inclusion of significant others, but not until model 3. This strengthens the impression of a gradually weakened association.

8 Single-level OLS regressions instead estimate no significant association between TC-parents and habituated aspirations in model 1, and a negative association between EU-born and aspirations in the last. Further models indicated that these differences are due to unequal distribution of EU-born students between schools which makes single level models confuse school characteristics – i.e. low average levels of habituated aspirations – with individual characteristics.

## References

[CR1] Ahmadi, F., Palm, I., & Ahmadi, N. (2016). *Mångfaldsbarometern 2016. FOU-rapport 44 [The Diversity Barometer 2016. FOU-report 44]*. Gävle: Gävle University Press.

[CR2] Appadurai A, Rao V, Walton M (2004). The capacity to aspire: Culture and the terms of recognition. Culture and public action.

[CR3] Archer L, Francis B (2006). Challenging classes? Exploring the role of social class within the identities and achievement of British Chinese pupils. Sociology.

[CR4] Bankston, C. L., Caldas, S. J., & Zhou, M. (1997). The academic achievement of Vietnamese American students: Ethnicity as social capital. *Sociological Focus*, *30*(1), 1–16. doi:10.1080/00380237.1997.10570679

[CR5] Bauer, P. C., & Riphahn, R. T. (2013). Institutional determinants of intergenerational education transmission — Comparing alternative mechanisms for natives and immigrants. *Labour Economics*, *25*, 110–122. doi:10.1016/j.labeco.2013.04.005

[CR6] Behtoui A (2006). Unequal opportunities : The impact of social capital and recruitment methods on immigrants and their children in the Swedish labour market.

[CR7] Behtoui A (2017). Social capital and the educational expectations of young people. European Educational Research Journal.

[CR8] Belot, M. V. K., & Hatton, T. J. (2012). Immigrant selection in the OECD. *Scandinavian Journal of Economics*, *114*(4), 1105–1128. doi:10.1111/j.1467-9442.2012.01721.x

[CR9] Boudon R (1974). Education, opportunity, and social inequality: Changing prospects in western society.

[CR10] Bourdieu P, Richardson JG (1986). The forms of capital. Handbook of theory and research for the sociology of education.

[CR11] Breen R, Jonsson JO (2005). Inequality of opportunity in comparative perspective: Recent research on educational attainment and social mobility. Annual Review of Sociology.

[CR12] Brinbaum Y, Cebolla-Boado H (2007). The school careers of ethnic minority youth in France: Success or disillusion?. Ethnicities.

[CR13] Buchmann C, Dalton B (2002). Interpersonal influences and educational aspirations in 12 countries: The importance of institutional context. Sociology of Education.

[CR14] Burger K (2016). Intergenerational transmission of education in Europe: Do more comprehensive education systems reduce social gradients in student achievement?. Research in Social Stratification and Mobility.

[CR15] Burgess, S., & Umaña-Aponte, M. (2011). *Raising your sights: The impact of friendship networks on educational aspirations *(Working paper no. 11/271). Bristol: Department of Economics, University of Bristol, UK, The Centre for Market and Public Organisation.

[CR16] Bursell M (2014). The multiple burdens of foreign-named men–evidence from a field experiment on gendered ethnic hiring discrimination in Sweden. European Sociological Review.

[CR17] Crozier G, Davies J (2006). Family matters: A discussion of the Bangladeshi and Pakistani extended family and community in supporting the children’s education. The Sociological Review.

[CR18] Crul M (2013). Snakes and ladders in educational systems: Access to higher education for second-generation Turks in Europe. Journal of Ethnic and Migration Studies.

[CR19] Crul M, Vermeulen H (2003). The second generation in Europe. The International Migration Review.

[CR20] Dahlstedt M, Hertzberg F (2014). In the name of liberation. European Education.

[CR21] Davies B, Bansel P (2007). Neoliberalism and education. International Journal of Qualitative Studies in Education.

[CR22] D’hondt, F., Van Praag, L., Van Houtte, M., & Stevens, P. A. (2016). The attitude-achievement paradox in Belgium: An examination of school attitudes of ethnic minority students. *Acta Sociologica*, *59*(3), 215–231. doi:10.1177/0001699316636944

[CR23] Engzell P (2016). Intergenerational persistence and ethnic disparities in education.

[CR24] European Commission. (2004). Final report of the expert group ‘Education for Entrepreneurship’. Retrieved from http://ec.europa.eu/DocsRoom/documents/2213/attachments/1/translations/en/renditions/pdf. Accessed 19 Sept 2017

[CR25] European Commission. (2006). Entrepreneurship action plan. Key action sheets. Key action 1 – Fostering entrepreneurial mindsets through school education. Retrieved from https://web.archive.org/web/20090423055605/http://ec.europa.eu/enterprise/entrepreneurship/action_plan/index.ht. http://ec.europa.eu/enterprise/entrepreneurship/action_plan/index.htm. (Accessed 23 Sept 2009)

[CR26] Feld SL (1981). The focused Organization of Social Ties. American Journal of Sociology.

[CR27] Feliciano C (2005). Educational selectivity in U.S. immigration: How do immigrants compare to those left behind?. Demography.

[CR28] Feliciano C, Lanuza YR (2016). The immigrant advantage in adolescent educational expectations. International Migration Review.

[CR29] Fernández-Reino, M. (2016). Immigrant optimism or anticipated discrimination? Explaining the first educational transition of ethnic minorities in England. *Research in Social Stratification and Mobility*. doi:10.1016/j.rssm.2016.08.007

[CR30] Flap, H., & Völker, B. (2004). Preface. In H. Flap, B. Völker (Eds.), *Creation and returns of social capital: A new research program*. London: Routledge.

[CR31] Flores-Gonzalez N (2005). Popularity versus respect: School structure, peer groups and Latino academic achievement. International Journal of Qualitative Studies in Education.

[CR32] Gabay-Egozi, L., Shavit, Y., & Yaish, M. (2015). Gender differences in fields of study: The role of significant others and rational choice motivations. *European Sociological Review*, *31*(3), 284–297. doi:10.1093/esr/jcu090

[CR33] Goyette K (2008). College for some to college for all: Social background, occupational expectations, and educational expectations over time. Social Science Research.

[CR34] Goyette K, Xie Y (1999). Educational expectations of Asian American youths: Determinants and ethnic differences. Sociology of Education.

[CR35] Griga D, Hadjar A (2013). Migrant background and higher education participation in Europe: The effect of the educational systems. European Sociological Review.

[CR36] Haller, A. O. (1968). On the Concept of Aspiration. *Rural Sociology, 33*(4), 484–487.

[CR37] Heath A, Brinbaum Y (2007). Guest editorial: Explaining ethnic inequalities in educational attainment. Ethnicities.

[CR38] Heath, A., & Brinbaum, Y. (2014). The comparative study of ethnic inequalities in Educaitonal careers. In A. Heath, Y. Brinbaum (Eds.), *Unequal attainments: Ethnic educational inequalities in ten western countries*, (p. 245). Oxford: British Academy.

[CR39] Heath, A., Rothon, C., & Kilpi, E. (2008). The second generation in Western Europe: Education, unemployment, and occupational attainment. *Annual Review of Sociology*, *34*, 211–235.

[CR40] Horn D (2009). Age of selection counts: A cross-country analysis of educational institutions. Educational Research and Evaluation.

[CR41] Ichou M (2014). Who they were there: Immigrants’ educational selectivity and their Children’s educational attainment. European Sociological Review.

[CR42] Jackson M (2012). Bold choices: How ethnic inequalities in educational attainment are suppressed. Oxford Review of Education.

[CR43] Jackson, M., & Jonsson, J. O. (2013). Why does inequality of educational opportunity vary across countries? Primary and secondary effects in comparative context. In M. Jackson (Ed.), *Determined to succeed? Performance versus choice in educational attainment*. Stanford, California: Stanford University Press.

[CR44] Jackson M, Jonsson JO, Rudolphi F (2012). Ethnic inequality in Choicedriven education systems: A longitudinal study of performance and choice in England and Sweden. Sociology of Education.

[CR45] Jonsson JO, Rudolphi F (2011). Weak performance—Strong determination: School achievement and educational choice among children of immigrants in Sweden. European Sociological Review.

[CR46] Kalmijn M, Kraaykamp G (2003). Dropout and downward mobility in the educational career: An event-history Anaylsis of ethnic schooling differences in the Netherlands. Educational Research and Evaluation.

[CR47] Kao G, Tienda M (1995). Optimism and achievement: The educational performance of immigrant youth. Social Science Quarterly.

[CR48] Kilpi-Jakonen E (2011). Continuation to upper secondary education in Finland: Children of immigrants and the majority compared. Acta Sociologica.

[CR49] Kristen, C., Reimer, D., & Kogan, I. (2008). Higher education entry of Turkish immigrant youth in Germany. *International Journal of Comparative Sociology*, *49*(2–3), 127–151. doi:10.1177/0020715208088909

[CR50] Lareau A (2002). Invisible inequality: Social class and childrearing in black families and white families. American Sociological Review.

[CR51] Lavrijsen J, Nicaise I (2015). Social inequalities in early school leaving: The role of educational institutions and the socioeconomic context. European Education.

[CR52] Le Donne N (2014). European variations in socioeconomic inequalities in Students’ cognitive achievement: The role of educational policies. European Sociological Review.

[CR53] Lin, N. (2001). *Social capital : A theory of social structure and action*. Cambridge; New York: Cambridge University Press.

[CR54] Madarasova Geckova, A., Tavel, P., Van Dijk, J. P., Abel, T., & Reijneveld, S. A. (2010). Factors associated with educational aspirations among adolescents: Cues to counteract socioeconomic differences? *BMC Public Health*, *10*, 154. doi:10.1186/1471-2458-10-154 10.1186/1471-2458-10-154 PMC2851685 20334644

[CR55] Marjoribanks K (1997). Family background, social and academic capital, and Adolescents’ aspirations: A Mediational analysis. Social Psychology of Education.

[CR56] Marjoribanks K (2002). Family background, individual and environmental influences on Adolescents’ aspirations. Educational Studies.

[CR57] Marjoribanks K (2010). Family background, individual and environmental influences, aspirations and young Adults’ educational attainment: A follow-up study. Educational Studies.

[CR58] Modood T (2004). Capitals, ethnic identity and educational qualifications. Cultural Trends.

[CR59] OECD (2013a). *PISA 2012 Results: Excellence through Equity (Volume II): Giving Every Student the Chance to Succeed, OECD Publishing, Paris. *http://dx.doi.org/10.1787/9789264201132-en

[CR60] OECD (2013b). *PISA 2012 Results: What Makes Schools Successful (Volume IV): Resources, Policies and Practices, OECD Publishing, Paris*. http://dx.doi.org/10.1787/9789264201156-en

[CR61] OECD (2016). *PISA 2015 Results (Volume I): Excellence and Equity in Education, OECD Publishing, Paris,* OECD Publishing. http://dx.doi.org/10.1787/9789264266490-en

[CR62] OECD & EU (2015). *Indicators of immigrant integration 2015: Settling in*. Paris: OECD Publishing. http://dx.doi.org/10.1787/9789264234024-en

[CR63] Raco M (2009). From expectations to aspirations: State modernisation, urban policy, and the existential politics of welfare in the UK. Political Geography.

[CR64] Salikutluk Z (2016). Why do immigrant students aim high? Explaining the aspiration–achievement paradox of immigrants in Germany. European Sociological Review.

[CR65] Sewell, W. H., Haller, A. O., & Portes, A. (1969). The educational and early occupational attainment process. *American Sociological Review*, *34*(1), 82–92.

[CR66] Sewell WH, Shah VP (1968). Parents’ education and Children’s educational aspirations and achievements. American Sociological Review.

[CR67] Shah, B., Dwyer, C., & Modood, T. (2010). Explaining educational achievement and career aspirations among young British Pakistanis: Mobilizing ‘ethnic capital’? *Sociology*, *44*(6), 1109–1127. doi:10.1177/0038038510381606

[CR68] Spohrer K (2011). Deconstructing ‘Aspiration’: UK policy debates and European policy trends. European Educational Research Journal.

[CR69] Spruyt B, De Keere K, Keppens G, Roggemans L, Van Droogenbroeck F (2014). What is it worth? An empirical investigation into attitudes towards education amongst youngsters following secondary education in Flanders. British Journal of Sociology of Education.

[CR70] Stahl G (2014). White working-class male narratives of ‘loyalty to self’ in discourses of aspiration. British Journal of Sociology of Education.

[CR71] Stanton-Salazar RD (2010). A social capital framework for the study of institutional agents and their role in the empowerment of low-status students and youth. Youth & Society.

[CR72] Stevens, P. A. J., Crul, M. J., Slootman, M. W., Clycq, N., Timmerman, C., & Houtte, M. V. (2014). The Netherlands. In P. A. J. Stevens, A. G. Dworkin (Eds.), *The Palgrave handbook of race and ethnic inequalities in education*. Basingstoke: Palgrave Macmillan UK.

[CR73] Teney, C., Devleeshouwer, P., & Hanquinet, L. (2013). Educational aspirations among ethnic minority youth in Brussels: Does the perception of ethnic discrimination in the labour market matter? A mixed-method approach. *Ethnicities*, *13*(5), 584–606. doi:10.1177/1468796812472009

[CR74] Thapar-Bjorkert S, Sanghera G (2010). Social capital, educational aspirations and young Pakistani Muslim men and women in Bradford, West Yorkshire. The Sociological Review.

[CR75] Tikkanen, J., Bledowski, P., & Felczak, J. (2015). Education systems as transition spaces. *International Journal of Qualitative Studies in Education*, *28*(3), 297–310. doi:10.1080/09518398.2014.987853

[CR76] Urban S (2012). University education as a compensation strategy among second-generation immigrants. International Migration Review.

[CR77] Van de Werfhorst HG, Mijs JJB (2010). Achievement inequality and the institutional structure of educational systems: A comparative perspective. Annual Review of Sociology.

[CR78] Van de Werfhorst HG, Van Tubergen F (2007). Ethnicity, schooling, and merit in the Netherlands. Ethnicities.

[CR79] Verkuyten M, Thijs J (2010). Racist victimization among children in The Netherlands: The effect of ethnic group and school. Ethnic and Racial Studies.

[CR80] Willis PE (1993). Learning to labour : How working class kids get working class jobs.

[CR81] Woelfel J, Haller AO (1971). Significant others, the self-reflexive act and the attitude formation process. American Sociological Review.

[CR82] Zhou M, Bankston CL (1994). Social capital and the adaptation of the second generation: The case of Vietnamese youth in New Orleans. The International Migration Review.

[CR83] Zipin, L., Sellar, S., Brennan, M., & Gale, T. (2013). Educating for futures in marginalized regions: A sociological framework for rethinking and researching aspirations. *Educational Philosophy and Theory*, *47*(3), 227–246. doi:10.1080/00131857.2013.839376

